# Simple Response Surface Methodology: Investigation on Advance Photocatalytic Oxidation of 4-Chlorophenoxyacetic Acid Using UV-Active ZnO Photocatalyst

**DOI:** 10.3390/ma8010339

**Published:** 2015-01-19

**Authors:** Kian Mun Lee, Sharifah Bee Abd Hamid

**Affiliations:** Nanotechnology & Catalysis Research Center (NANOCAT), Institute of Postgraduate Studies, University of Malaya, 50603 Kuala Lumpur, Malaysia; E-Mail: leekianmun@um.edu.my

**Keywords:** zinc oxide, central composite design, response surface methodology, 4-chlorophenoxyacetic acid, photocatalytic degradation

## Abstract

The performance of advance photocatalytic degradation of 4-chlorophenoxyacetic acid (4-CPA) strongly depends on photocatalyst dosage, initial concentration and initial pH. In the present study, a simple response surface methodology (RSM) was applied to investigate the interaction between these three independent factors. Thus, the photocatalytic degradation of 4-CPA in aqueous medium assisted by ultraviolet-active ZnO photocatalyst was systematically investigated. This study aims to determine the optimum processing parameters to maximize 4-CPA degradation. Based on the results obtained, it was found that a maximum of 91% of 4-CPA was successfully degraded under optimal conditions (0.02 g ZnO dosage, 20.00 mg/L of 4-CPA and pH 7.71). All the experimental data showed good agreement with the predicted results obtained from statistical analysis.

## 1. Introduction

Recently, advanced oxidation processes (AOPs) have been studied extensively for both recalcitrant wastewater and dye wastewater. The reason is mainly attributed to a high capability to mineralize a wide range of recalcitrant contaminants and organic dye compounds via AOPs. In this case, AOPs have emerged as an efficient and cost-effective method in wastewater treatment. Theoretically, AOPs are based on the generation of highly reactive species, such as H_2_O_2_, ·OH, ·O_2_^−^ and O_3_ for completing destruction of refractory organic compounds, including xylenols, pyridine, methylpyrrolidone, thiophene, *etc.* [1,2,3,4,5,6]. Among viable AOPs approaches, the use of heterogeneous photocatalysis system by employing semiconductor photocatalyst has become one of the most promising methods and has high potential to secure our green environment [7,8,9,10]. In order to bring the advanced heterogeneous photocatalysis system to the point of commercial readiness and establish a green economy for our next generation, substantial research on the development of an efficient semiconductor for wastewater treatment has been developed lately.

To date, zinc oxide (ZnO) has emerged as the leading candidate in the advanced heterogeneous photocatalysis system because of its unique characteristics, such as long-term photo-stability, excellent chemical stability, non-toxicity in nature, and outstanding charge transport property. It is a well-known fact that a ZnO photocatalyst has almost the same band gap energy (3.2 eV) as titanium dioxide (TiO_2_) and exhibited strong oxidation property under UV irradiation. Thus, ZnO can be acted as potential candidate in total mineralization of environmental contaminants, especially in wastewater treatment application [11,12,13,14,15]. Moreover, ZnO is relatively cost-effective compared to TiO_2_ whereby the usage of TiO_2_ is uneconomical for large scale water treatment operations [16]. Nevertheless, the greatest advantage of ZnO over TiO_2_ is the ability to absorb a wide range of UV spectrum with the corresponding threshold of 425 nm [17].

Nowadays, advanced crop protection technology has been received lots of scientific interest, which includes all pesticides, herbicides, insecticides, fungicides, as well as biotechnology products. This crop protection technology helps control the thousands of weed species, harmful insects and numerous plant diseases that afflict crops. However, the use of herbicides and pesticides in agricultural field on a large scale created severe environmental problems, especially water pollution. The most crucial issue is that all herbicides and pesticides compounds are chemically stable as well as resist to biodegradation. Moreover, the toxicity possessed by these organics may cause health problems. The widespread use of chlorophenoxy herbicides for agricultural purposes has raised public concern due to the accumulation of disposed residual in natural waters [18]. 4-chlorophenoxyacetic acid (4-CPA) is a widely used herbicide in controlling the sprout formation in mung beans [19]. A few reports have been carried out in removing 4-CPA [19,20,21], however, those studies only dealing with one-factor-at-a-time while holding others parameters constant. The optimization by this method is not adequate due to the combination effect of the parameters was not considered. Moreover, more time is needed and the true optimum conditions are hard to predict [22,23]. This will indirectly increase the cost of the overall process [24].

In order to overcome this problem, the optimization studies have been carried out by response surface methodology (RSM). RSM is a combination of statistical and mathematical method for optimization study in a complicated process [25]. RSM gives a lot of information from a small number of experiments compared to conventional methods. In addition, this statistical design of experiments taking into account the interaction effects between the studied parameters and can determine the combination of levels in order to optimize the process more accurately [26,27,28,29]. A central composite design (CCD) based on RSM was successfully applied in the optimization of photodegradation of various organics [30,31,32,33,34]. To the best of our knowledge, the literature regarding to the advance photocatalytic oxidation of 4-CPA using UV-active ZnO photocatalyst is still not available yet. Therefore, the well-designed and controlled of the critical parameters (ZnO loading, initial concentration of 4-CPA and initial pH) in heterogeneous photocatalysis system have been carried out using a simple RSM in order to maximize 4-CPA removal.

## 2. Materials and Methods

### 2.1. Materials

Zinc oxide (99%) and 4-chlorophenoxyacetic acid (4-CPA) (99%) in this study were purchased from Merck (Darmstadt, Germany). The chemicals were used as received without any purification. Deionized water (18.2 MΩ) was used throughout the studies to prepare the stock solutions and working solutions of 4-CPA. The specific surface area of ZnO particles was determined by the static BET method using a Thermo Finnigan Sorptomatic 1990 series analyzer (Thermo Fisher Scientific Inc., Milan, Italy). The band gap energy of ZnO was recorded by a Perkin Elmer Lambda 35 UV-vis-NIR spectrometer (Perkin Elmer, Waltham, MA, USA) equipped with an integrating sphere at room temperature. The particle size of ZnO was measured on the Nanophox facility (Sympatec, Clausthal-Zellerfeld, Germany).

### 2.2. Photocatalytic Degradation Studies

The photocatalytic studies were carried out in a rectangular photoreactor with five parallel quartz vessels. In a typical experiment, the ZnO photocatalyst was suspended in 100 mL of 4-CPA solution and was irradiated with a 96 W UV-A lamp with maximum wavelength of 365 nm. The initial pH of the solution was adjusted by a small amount of 0.01 M HNO_3_ or 0.01 M NaOH to the desired pH. The solution was allowed to stir for 20 min in the dark to attain adsorption equilibrium before irradiation. During irradiation, air was bubbled into the reaction medium to ensure a constant supply of oxygen (2 L/min). Stirring was applied to ensure a complete suspension of catalyst particles. The residual of 4-CPA in the test solution was filtered by using 0.22 μm Nylon filter to remove the ZnO particles. The concentration of 4-CPA in test samples was determined at λ_max_ = 279 nm by a Perkin Elmer Lambda 35 UV-Vis spectrophotometer (Perkin Elmer, Waltham, MA, USA). All photodegradation experiments were done in triplicate to ensure the reproducibility of the experimental results. The Total Organic Carbon (TOC) content of degradation of 4-CPA was carried out (In House, based on HACH method) to evaluate the extent of mineralization.

### 2.3. Response Surface Methodology

In the optimization study, RSM was used to optimize the three parameters (ZnO loading, initial concentration of 4-CPA and initial pH). The three parameters were selected as independent variables while the degradation percentage of 4-CPA was the output response variable. Other factors such as stirring rate, temperature, light intensity and oxygen supply were held constant. Table 1 shows the ranges and level of independent variables. Central composite design (CCD) was chosen to investigate the combined effect of the three independent variables by 20 sets of experiments, including six replications at the center points. The experimental values of 4-CPA degradation percentage under various experimental conditions are shown in Table 2. Design Expert V.8.0.6 (Stat-Ease Inc., Minneapolis, MN, USA) was adopted to describe the response surface. In order to check the accuracy of the fitted model, a series of statistical analysis such as the normal plot, the residual analysis, the main and interaction effects, the contour plot and analysis of variance (ANOVA) was examined.

**Table 1:** Independent variables and experimental range for degradation of 4-chlorophenoxyacetic acid (4-CPA).

Factors	Unit	Symbol	Range				
			−2	−1	0	+1	+2
ZnO loading	g	*x*_1_	0.01	0.02	0.03	0.04	0.05
Initial 4-CPA concentration	mg/L	*x*_2_	10.00	20.00	30.00	40.00	50.00
pH	*–*	*x*_3_	5.00	6.00	7.00	8.00	9.00

**Table 2:** Central composite design with predictive values and their experimental results.

Run	Experimental conditions			4-CPA degradation (%)		
	*x*_1_ ZnO loading (g)	*x*_2_ Initial 4-CPA concentration (mg/L)	*x*_3_ pH	Experimental	Predictive	Residual
1	0.02	20.00	6.00	80.32	80.07	0.25
2	0.04	20.00	6.00	76.49	77.31	−0.82
3	0.02	40.00	6.00	73.03	73.25	−0.22
4	0.04	40.00	6.00	66.41	66.26	0.15
5	0.02	20.00	8.00	99.32	99.99	−0.67
6	0.04	20.00	8.00	98.89	99.19	−0.30
7	0.02	40.00	8.00	90.73	90.43	0.30
8	0.04	40.00	8.00	84.64	85.41	−0.77
9	0.01	30.00	7.00	84.43	84.52	−0.094
10	0.05	30.00	7.00	77.36	76.75	0.61
11	0.03	10.00	7.00	100.00	99.49	0.51
12	0.03	50.00	7.00	78.91	78.90	0.014
13	0.03	30.00	5.00	51.50	51.44	0.059
14	0.03	30.00	9.00	90.97	90.51	0.46
15	0.03	30.00	7.00	95.32	95.15	0.17
16	0.03	30.00	7.00	94.88	95.15	−0.27
17	0.03	30.00	7.00	93.02	95.15	−2.13
18	0.03	30.00	7.00	95.69	95.15	0.54
19	0.03	30.00	7.00	95.50	95.15	0.35
20	0.03	30.00	7.00	97.01	95.15	1.86

## 3. Results and Discussion

### 3.1. Preliminary Experiments

**Figure 1:**
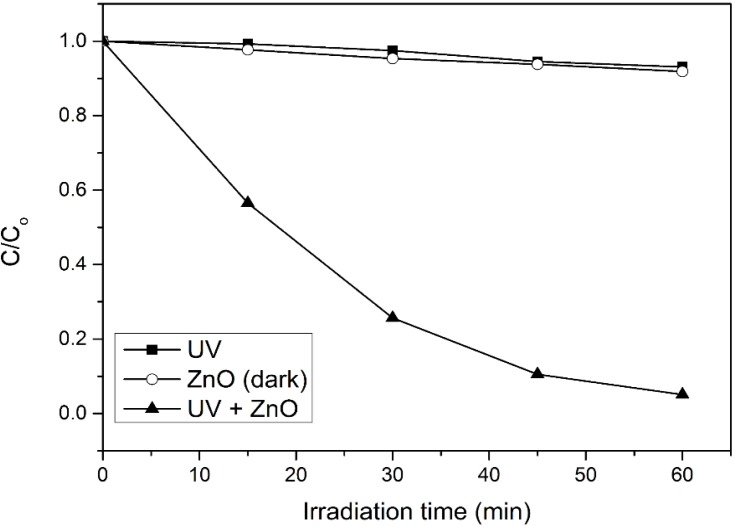
Photocatalytic degradation of 4-CPA under various conditions. Conditions: ZnO loading = 0.03 g; [4-CPA] = 30 mg/L; pH = 7.

**Figure 2:**
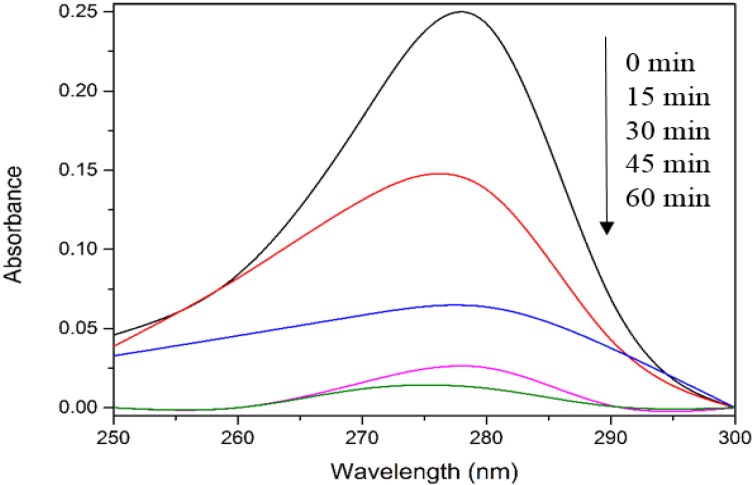
UV-vis spectra of 4-CPA during photocatalytic degradation process. Conditions: ZnO loading = 0.03 g; [4-CPA] = 30 mg/L; pH = 7.

In order to evaluate the significant role of photocatalysis in the photodegradation process, photolysis and catalyst adsorption in dark condition were carried out. As shown in Figure 1, only 7% of 4-CPA was photolyzed by UV irradiation. The absorption of 4-CPA by ZnO photocatalyst was insignificant (8.2%). However, 94.9% of 4-CPA was degraded in the presence of ZnO under UV irradiation for an hour. This indicated the synergistic effect between ZnO photocatalyst and UV light for the photocatalytic degradation to work efficiently. Figure 2 depicts the UV-vis spectra of 4-CPA during photocatalysis process. As evident from Figure 2, no obvious peak shift was observed.

### 3.2. Model Fitting and Statistically Analysis

In order to optimize the reaction conditions of 4-CPA degradation, CCD with a total number of 20 experiments was applied for the response surface modeling (Table 2). The experimental and predicted responses are shown as well. The software suggested quadratic model as shown in Table 3. Table 4 shows the ANOVA for response surface of quadratic model. The lack of fit indicates the variation of data around the fitted model and it will shows significant if the data does not fit well with the model [25]. The ANOVA implies that the model is significant with the *F* value of 301.23. There is only a 0.01% chance that a “Model *F*-Value” could occur due to noise. Moreover, the *p* value (<0.0001) is less than 0.05, which indicates the model terms are highly significant. It should be noted that values greater than 0.1000 indicate the model terms are not significant [35,36].

**Table 3:** Sequential model fitting for 4-CPA removal.

Source	Sum of squares	Degree of freedom	Mean square	*F* Value	*p* Value	Remark
Mean	1.487 × 10^5^	1	1.487 × 10^5^	–	–	–
Linear	2011.02	3	670.34	9.85	0.0006	–
2 FI	14.60	3	4.87	0.059	0.9805	–
Quadratic	1063.20	3	354.40	311.05	<0.0001	Suggested
Cubic	1.93	4	0.48	0.31	0.8637	Aliased
Residual	9.46	6	1.58	–	–	–
Total	1.518 × 10^5^	20	7589.07	–	–	–

**Table 4:** ANOVA for the response surface quadratic model.

Source	Sum of squares	DF	Mean square	*F* Value	*p* Value	Suggestion
Model	3088.82	9	343.20	301.23	<0.0001	Significant
*x*_1_	60.49	1	60.49	53.09	<0.0001	–
*x*_2_	424.26	1	424.26	372.37	<0.0001	–
*x*_3_	1526.27	1	1526.27	1339.59	<0.0001	–
*x*_1_*x*_2_	8.93	1	8.93	7.83	0.0188	–
*x*_1_*x*_3_	1.93	1	1.93	1.69	0.2222	–
*x*_2_*x*_3_	3.74	1	3.74	3.28	0.1001	–
*x*_1_^2^	331.08	1	331.08	290.58	<0.0001	–
*x*_2_^2^	55.73	1	55.73	48.91	<0.0001	–
*x*_3_^2^	918.39	1	918.39	806.06	<0.0001	–
Residual	11.39	10	1.14	–	–	–
Lack of fit	2.93	5	0.59	0.35	0.8658	Not significant
Pure error	8.47	5	1.69	–	–	–
Std. dev. ^a^	1.07	–	*R*^2^	0.9963	–	–
Mean	86.22	–	Adj. *R*^2 b^	0.9930	–	–
C.V. ^c^	1.24	–	Pred. *R*^2 d^	0.9889	–	–
PRESS	34.47	–	Adeq. precision ^e^	64.317	–	–

^a^ Standard deviation. ^b^ Adjusted *R*^2^. ^c^ Coefficient of variation. ^d^ Predicted *R*^2^. ^e^ Adequate precision.

The lack of fit value of 0.35 confirms the lack of fit is not significant relative to the pure error when *p* value is 0.8658, >0.05. The non-significant lack of fit shows good predictability of the model. The coefficient of variation (C.V. = 1.24) is low, indicated high precision and good reliability of the experimental values [37]. In addition, the “Pred. *R*^2^” of 0.9889 is in reasonable agreement with the “Adj. *R*^2^” of 0.9930 (within 0.2), which also implies good predictability of the model [38,39]. Adequate precision compares the range of predicted values at the design points to the average prediction error [40,41]. The adequate precision of 64.317 in this study, which is well above 4 indicates adequate model discrimination.

**Figure 3:**
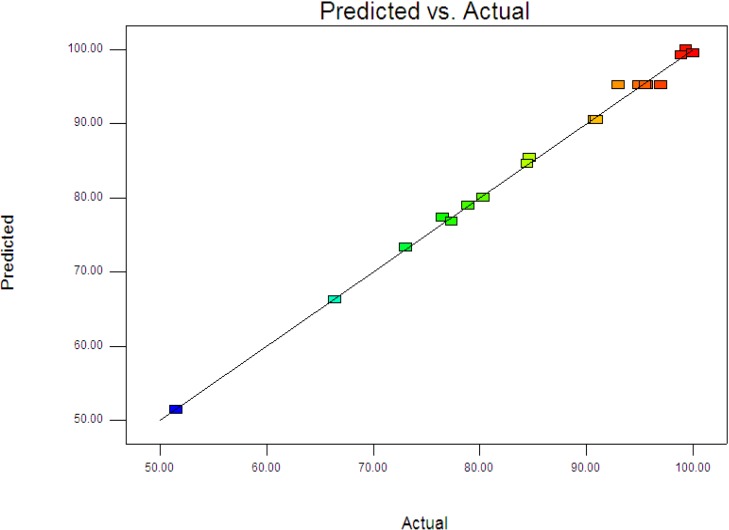
Predicted *vs.* actual values for photodegradation of 4-CPA.

**Figure 4:**
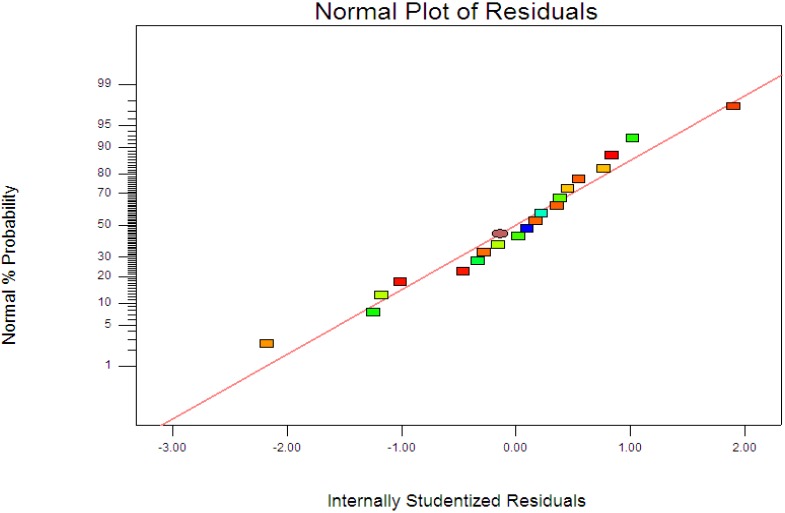
Normal probability plots of the residuals.

**Figure 5:**
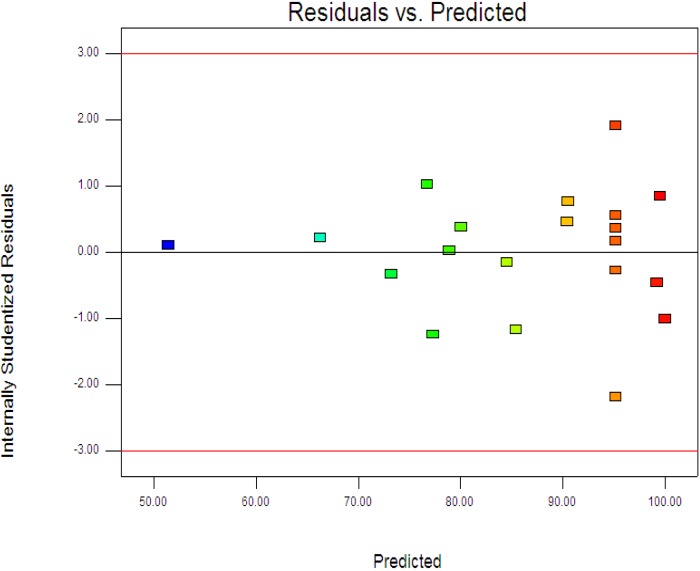
Plot of the residuals against the predicted response.

From the experimental results (Table 5), an empirical second-order polynomial equation was established and was written in terms of coded factors as follows:

*y* = 95.15 − 1.94*x*_1_ − 5.15*x*_2_ + 9.77*x*_3_ − 3.63*x*_1_*x*_2_ − 1.49*x*_1_*x*_3_ − 6.04*x*_2_*x*_3_ − 1.06*x*_1_^2^ + 0.49*x*_2_^2^ − 0.68*x*_3_^2^ (1)

where *y* is the percentage degradation of 4-CPA (%), *x*_1_, *x*_2_ and *x*_3_ are terms for the coded values of ZnO loading, initial concentration of 4-CPA and pH, respectively. Figure 3 displays the experimental and predictive values for 4-CPA degradation. It can be seen that the high correlation between the experimental data and predicted values (*R*^2^ = 0.9963) showed the data fit well with the model in the range studied. On the other hand, residuals analysis was carried out in order to confirm the adequacy of the model. This was done by observing the normal probability plot of the residuals (Figure 4) and the plot of the residuals *vs.* the predicted response (Figure 5). From Figure 4, the residuals were fall on a straight line suggested the errors are distributed normally [42]. Furthermore, structureless pattern in the plot of residuals *vs.* the predicted response indicated the model is adequate and the model does not show any violation of the independence or constant variance assumption [40].

**Table 5:** Coefficient of regression model and their significance.

Factor	Coefficient estimate	Degree of freedom	Standard error	*F* Value	95% Confidence interval low	95% Confidence interval high	*p* Value
Intercept	95.15	1	0.43	–	94.20	96.10	–
*x*_1_	−1.94	1	0.27	53.09	−2.54	−1.35	<0.0001
*x*_2_	−5.15	1	0.27	372.37	−5.74	−4.55	<0.0001
*x*_3_	9.77	1	0.27	1339.59	9.17	10.36	<0.0001
*x*_1_^2^	−1.06	1	0.38	7.83	−1.90	−0.22	0.0188
*x*_2_^2^	0.49	1	0.38	1.69	−0.35	1.33	0.2222
*x*_3_^2^	−0.68	1	0.38	3.28	−1.52	0.16	0.1001
*x*_1_*x*_2_	−3.63	1	0.21	290.58	−4.10	−3.15	<0.0001
*x*_1_*x*_3_	−1.49	1	0.21	48.91	−1.96	−1.01	<0.0001
*x*_2_*x*_3_	−6.04	1	0.21	806.06	−6.52	−5.57	<0.0001

### 3.3. Response Surface Analysis

**Figure 6:**
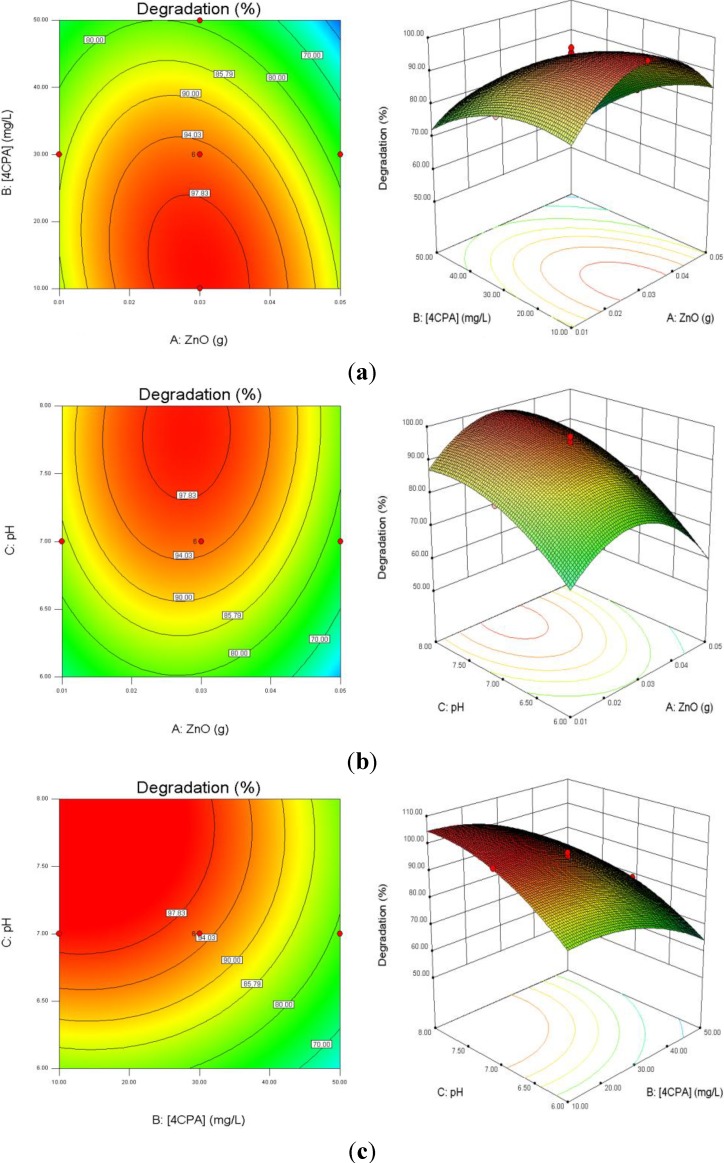
Effects of ZnO loading, initial concentration of 4-CPA and pH on the degradation percentage of 4-CPA. (**a**) pH was kept constant at 7.0; (**b**) 4-CPA initial concentration was kept constant at 30 mg/L; (**c**) ZnO loading was kept constant at 0.03 g.

Figure 6a depicts the influence of ZnO loading and initial 4-CPA concentration on the degradation efficiency while keeping pH at 7.00. As illustrated in the plots, the degradation percentage increased with increasing catalyst dosage up to an optimum mass (0.30 g) and then decreased with excessive ZnO. This is based on the fact that an increase in the catalyst dosage will enhance the number of active site on the catalyst surface, which in turn increased the number of hydroxyl and superoxide radicals responsible for the degradation of 4-CPA molecules [43]. Further enhancement in the catalyst concentration resulted in lower percentage of degradation. This may be due to the solution becomes opaque which reduces the penetration of UV light into the solution and consequently lowers the degradation percentage [44]. On the other hand, the removal percentage of 4-CPA was lower when the concentration increased from 10 to 50 mg/L. This can be explained by as the concentration of substrate increases, more and more pollutant molecules are adsorbed on the catalyst surface. However, the catalyst dosage, light intensity and irradiation period are remaining constant. Hence, the numbers of hydroxyl radicals formed on the catalyst surface are constant as well. Consequently, the hydroxyl radicals formed are insufficient to degrade substrate molecules at higher concentrations, which decreased the degradation efficiency [45].

Figure 6b presents the interaction effect of ZnO loading and initial solution pH on the removal of 4-CPA. It is evident that the removal increased as the pH and the amount of ZnO loaded up to an optimum value. The degradation rate decreased after achieved the optimal points. Higher catalyst loading does not favor the degradation process as it facilitates particle agglomeration leading to a reduction in catalyst surface area available for light absorption and pollutant adsorption, which in turn, reduced the photocatalytic efficiency [46]. The pK_a_ of 4-CPA is 3.56 and the point of zero charge (pH_zpc_) of ZnO is 9.0. Therefore, the 4-CPA is negatively charged above its pK_a_ value. As expected, the pH that shows optimal degradation must fall in between pK_a_ < pH < pH_zpc_, due to the electrostatic interaction among the anions of 4-CPA and positively charged catalyst surface is enhanced [47].

The effect of initial concentration of 4-CPA and pH values on the removal of 4-CPA are shown in Figure 6c. It is obvious that increasing the 4-CPA concentration adversely affect the removal efficiency. This phenomenon may be due to the screening effect by 4-CPA molecules which reduced light penetration into the solution. Thus, the photoactivated volume is reduced, which lowers the percentage of degradation [48]. The competition between the pollutant molecules and the generated intermediates for hydroxyl radicals also reduced the degradation rate at higher concentration [49]. In addition, the percentage removal was low at lower medium pH regardless 4-CPA concentration due to substantial loss of ZnO particles [50].

### 3.4. Process Optimization and Confirmation

**Figure 7:**
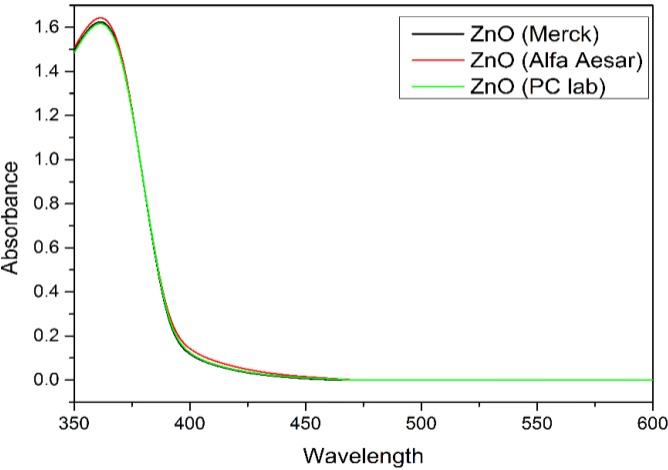
UV-Vis spectra of three different types of ZnO photocatalyst.

The goal of the optimization process is to achieve maximum degradation of 4-CPA by ZnO photocatalyst under UV irradiation. Therefore, the catalyst loading was set to minimum value, the concentration of 4-CPA was set to maximum value and the pH of the solution was set within the studied range, in order to obtain maximum degradation efficiency of 4-CPA. The optimum values of the influencing factors for the maximum 4-CPA removal under these circumstances are 0.02 g ZnO dosage, 20.00 mg/L 4-CPA and at pH 7.71 which gave 90.94% 4-CPA removal. Consequently, experiment was conducted in accordance to the optimized parameters and it showed 91.33% of 4-CPA removal. The good agreement between the predictive results and experimental results indicated that CCD design is feasible to optimize the degradation of 4-CPA. The photoactivity of ZnO (Merck) was compared with two other types of commercial ZnO (Alfa Aesar and PC lab) and the results were summarized in Table 6. It is obvious that the photocatalytic activity of ZnO powder was influenced by the surface area, where ZnO from Merck has the highest photoactivity compared to Alfa Aesar ZnO and PC lab ZnO. The UV-Vis spectra of ZnO photocatalyst (Merck, Alfa Aesar and PC lab) were shown in Figure 7.

**Table 6:** Characteristic of three types of commercial ZnO.

ZnO type	Characteristic			4-CPA degradation (%)
	Surface area (m^2^/g)	Particle size (μm)	Band gap (eV)	
Merck	3.3	0.4–0.5	3.02	91.33
PC lab	2.3	0.1–0.6	3.07	78.42
Alfa Aesar	1.5	0.1–0.3	3.04	76.23

### 3.5. Kinetic and Mineralization Studies

The reaction rate of 4-CPA in the presence of ZnO photocatalyst under UV irradiation was evaluated at different concentrations. The Langmuir-Hinshelwood (L-H) kinetic model was used to explain the kinetics of heterogeneous photocatalytic processes [51]:

(2) $$r_{1}=\;-\frac{dC}{dt}=\frac{K_{1}K_{2}C}{1+K_{2}K_{0}}=k_{1}C$$

where *C*_0_ is the initial concentration of 4-CPA (mg·L^−1^), *C* is the 4-CPA concentration at time, *t*, *k*_1_ is the pseudo first-order rate constant, *K*_1_ is the surface reaction rate constant (mg·L^−1^·min^−1^), *K*_2_ is the Langmuir-Hinshelwood adsorption equilibrium constant (L·mg^−1^). Integration of Equation (2) gives Equation (3):

(3) $$\text{ln}\frac{C}{C_{0}}+K_{2}\left(C_{0}-C\right)=K_{1}K_{2}t$$

When the solution is highly diluted, *C* (mol·L^−1^) < 10^−3^ [52], the term *K*_2_*C* becomes < 1, thus the denominator of Equation (2) is neglected and the reaction is essentially an apparent first-order reaction (Equation (4)):

(4) $$r_{1}=\;-\frac{dC}{dt}=\;K_{1}K_{2}C=k_{1}C$$

where *k*_1_ is the rate constant of a pseudo first-order reaction. Thus, Equation (3) can be simplified to a first-order reaction when *C*_0_ is very small gives Equation (5):

(5) $$\text{ln}\frac{C_{0}}{C}=k_{1}t$$

**Figure 8:**
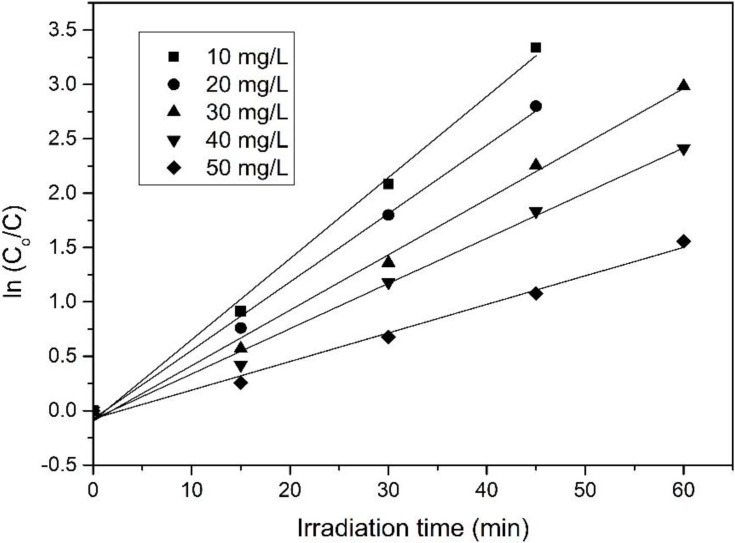
First-order rate graph of photocatalytic degradation on 4-CPA under UV illumination.

By plotting graph ln(*C*_0_/*C*) *vs.* *t*, the first-order rate constant (*k*_1_) can be determined from the slope of the straight line graph (Figure 8) and the values were tabulated in Table 7. A high regression coefficients (*R*^2^ > 0.99) implying that the results fitted well with the corresponding reaction kinetic. The rate of decomposition was the highest at low concentration of 4-CPA (10 mg/L) and gradually decreased at higher concentrations [53,54,55].

**Table 7:** First-order reaction rate, *k*_1_ of photodecompositon of 4-CPA.

Initial concentration of 4-CPA (mg/L)	First-Order rate constant, *k*_1_ (×10^−2^ min^−1^)	*R*^2^
10	7.46	0.9952
20	6.29	0.9957
30	5.10	0.9953
40	4.16	0.9940
50	2.63	0.9905

**Figure 9:**
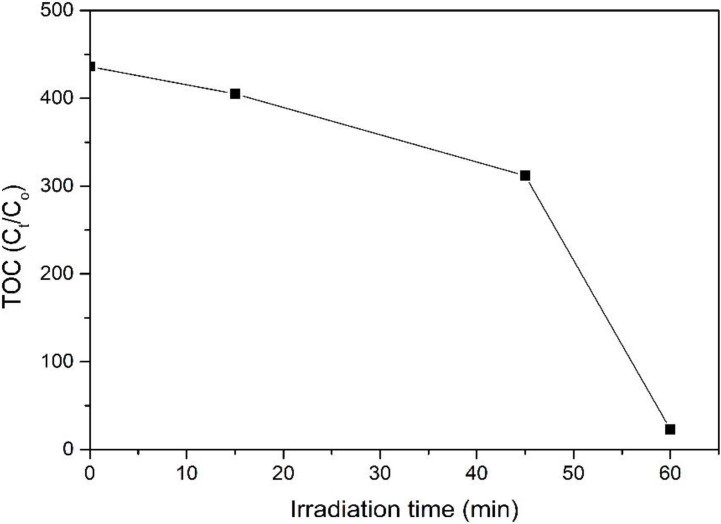
Relative total organic carbon (TOC) decay of 4-CPA over ZnO photocatalyst.

Figure 9 illustrates the relative TOC decay of 4-CPA over ZnO photocatalyst under UV irradiation. The mineralization rate increases with increasing irradiation time. It can be seen that the UV/ZnO system could mineralize 4-CPA effectively, achieved 94.7% mineralization with only 1 h, indicating most of the 4-CPA was mineralized during the photocatalytic degradation process.

## 4. Conclusions

The photocatalytic degradation of 4-CPA in aqueous medium has been examined by using ZnO under UV irradiation. A multivariate experimental design was employed to develop a quadratic model as the functional relationship between the studied factors, such as photocatalyst dosage, initial concentration of 4-CPA and pH of the solution to determine the optimum degradation percentage of 4-CPA. The removal of 4-CPA achieved 91.33% under optimal conditions (0.02 g ZnO loading, 20.00 mg/L 4CPA and at pH 7.71).
